# The complete chloroplast genome and phylogenetic analysis of *Manglietia ventii* (Magnoliaceae)

**DOI:** 10.1080/23802359.2021.2018951

**Published:** 2022-01-10

**Authors:** Yuchang Wang, Dawei Wang

**Affiliations:** Key Laboratory for Forest Resources Conservation and Utilization in the Southwest Mountains of China Ministry of Education, Southwest Forestry University, Kunming, China

**Keywords:** *Manglietia ventii*, genome sequence, chloroplast genome

## Abstract

*Manglietia ventii* is a highly ornamental timber tree species that is listed as endangered on the IUCN Red List. This study was conducted on the complete chloroplast genome of *M. ventii.* The length of the chloroplast genome is 159,950 bp with GC content as 39.3%. One hundred and thirty-one functional genes were identified in the genome, which included 86 protein-coding genes (PCGs), 37 tRNA genes, and eight rRNA genes. The phylogenetic analysis indicated that *M. ventii* is most closely related to *M. megaphylla* and *M. aromatica*, and the study provides new insights into the evolution of the Magnoliaceae.

*Manglietia ventii* is a species of timber with high ornamental value, which belongs to Magnoliaceae. The species is endemic to China and occurs in a small area in southeast Yunnan. *M. ventii* generally grows in valley woods at 800–1200 m (Flora of China Editorial Committee 1996). Due to the over-utilization of resources, the increasing destruction of primary forests, and the decline of the natural reproductive capacity, some Magnoliaceae are on the verge of extinction or even extinct (Cicuzza et al. 2007; Sima and Lu 2009). *M. ventii* is listed as an endangered species in the Red List, with no more than 100 adult individuals presumably in the wild (IUCN 2014). Here, its complete chloroplast genome was sequenced, assembled, and analyzed, which will provide favorable tools for researching the phylogenetic and origination of different Magnoliaceae.

Mature, healthy, and fresh leaves from *M. ventii* were collected from Kunming (102°45′E, 25°3′N, 1900 m), Yunnan province of China. The voucher specimen was deposited in the herbarium of Southwest Forestry University (http://www.swfu.edu.cn/, contact person: Jingyu Peng, Peng.Jingyu@outlook.com, accession number: SWFU-MAG-MMV-7739). (The experimental samples for this study were cultivated materials, collected from the campus of Southwest Forestry University and brought back to the studio for experimental studies, and the experimental materials were collected in accordance with relevant institutional, national and international guidelines and regulations. The samples were collected without causing harm to the plant habitat or the trees themselves). Extraction of total genomic DNA using a modified CTAB method and sequencing by Annoroad Gene Technology (Beijing, China) on Illumina Novaseq 6000 platform (Yang et al. 2014). The clean data were annotated in Geneious v8.1.3 software (Geneious, Auckland, New Zealand) using the *Manglietia longirostrata* (GenBank accession number MT584886) complete chloroplast genome as a reference. GetOrganelle v1.6.0 software (Jin et al. 2020) was used to assemble the complete chloroplast genome. The successfully annotated genome was submitted to GenBank (accession number: MW415421).

The complete chloroplast genome is a typical loop-like molecule of a four-part structure of 159,950 bp in length, which contains a small single-copy (SSC) replication region of 18,800 bp and a large single-copy (LSC) replication region of 88,008 bp linked by two inverted repeats regions (IRa and IRb) of 26,571 bp. The overall GC content is 39.3%, while the GC content of SSC, LSC, and IR regions is 34.0%, 38.0%, and 43.0%, respectively. There are 86 protein-coding genes (PCGs), eight rRNA, and 37 tRNA. Among these genes, nine PCGs (*petB*, *atpF*, *ndhA*, two *ndhB*, *rpoC1*, two *rpl2*, and *rps16*) and all tRNAs contain one intron, whereas four (two *rps12*, *clpP*, and *ycf3)* have two introns.

To reveal the evolutionary position of *M. ventii* among Magnoliaceae, *M. ventii* and the previously reported 45 cp genomes were aligned to the chloroplast genome matrix using MAFFT v7.487 (Katoh and Standley 2013; Chen et al. 2020; Sima et al. 2021) for ML analysis was based on the TVM + F+R2 model (Kalyaanamoorthy et al. 2017), using 1000 bootstrap replicates (Gao et al. 2018). Based on the system of Magnoliaceae by Sima and Lu (2012), the phylogenetic analysis indicates that *M. ventii* is most closely related to *M. megaphylla* and *M. aromatica* (Figure 1). Although *M. ventii* is separate from *M. decidua*, they still have a very strong support. *Liriodendron chinense* and *Liriodendron tulipifera* of the subfamily Liriodendroideae were served as the outgroup. All genera mentioned for this analysis are monophyletic according to the system of Magnoliaceae by Sima and Lu (2012). These results will provide valuable information for the phylogenetic and evolutionary study of the Magnoliaceae.

**Figure 1.**
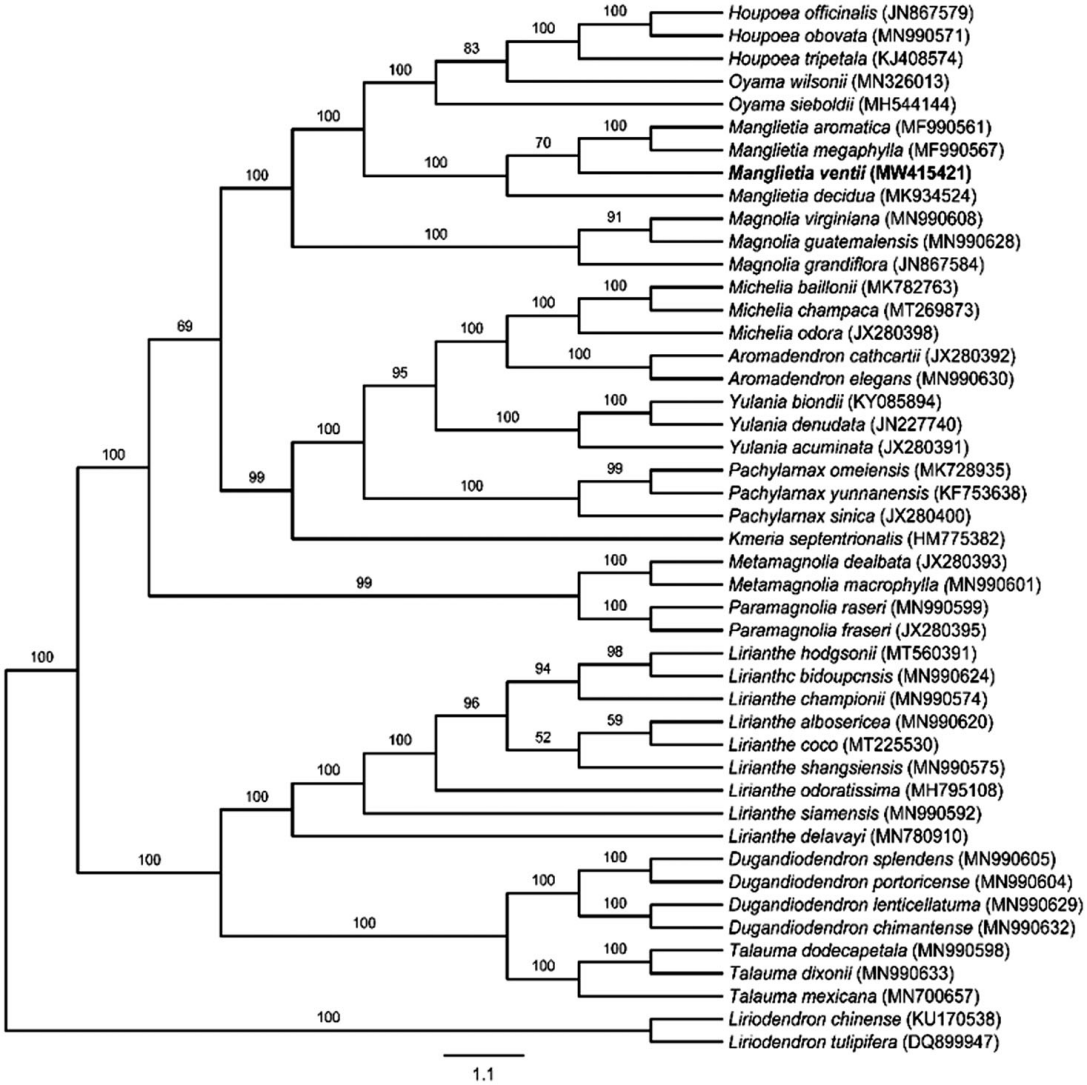
Maximum-likelihood (ML) phylogenetic tree based on complete cp genomes. Numbers close to each node are bootstrap support values.

## Data Availability

The data that support the findings of this study are available in NCBI GenBank (https://www.ncbi.nlm.nih.gov/) under the GenBank accession number MW415421. The associated BioProject, SRA, and BioSample numbers are PRJNA770098, SRR16352012, and SAMN22209219, respectively.
